# Mechanism and Modulation of SidE Family Proteins in the Pathogenesis of *Legionella pneumophila*

**DOI:** 10.3390/pathogens12040629

**Published:** 2023-04-21

**Authors:** Yongchao Xie, Yi Zhang, Yong Wang, Yue Feng

**Affiliations:** 1 Beijing Advanced Innovation Center for Soft Matter Science and Engineering, Beijing Key Laboratory of Bioprocess, State Key Laboratory of Chemical Resource Engineering, College of Life Science and Technology, Beijing University of Chemical Technology, Beijing 100029, China; 2 State Key Laboratory of Crop Biology, College of Life Sciences, Shandong Agricultural University, Tai’an 271002, China

**Keywords:** *Legionella pneumophila*, SidE family, effector, PR-ubiquitination, host-pathogen interaction

## Abstract

*Legionella pneumophila* is the causative agent of *Legionnaires’* disease, causing fever and lung infection, with a death rate up to 15% in severe cases. In the process of infection, *Legionella pneumophila* secretes over 330 effectors into host cell via the Dot/Icm type IV secretion system to modulate multiple host cellular physiological processes, thereby changing the environment of the host cell and promoting the growth and propagation of the bacterium. Among these effector proteins, SidE family proteins from *Legionella pneumophila* catalyze a non-canonical ubiquitination reaction, which combines mono-ADP-ribosylation and phosphodiesterase activities together to attach ubiquitin onto substrates. Meanwhile, the activity of SidE family proteins is also under multiple modulations by other effectors. Herein we summarize the key insights into recent studies in this area, emphasizing the tight link between the modular structure of SidE family proteins and the pathogen virulence as well as the fundamental mechanism and modulation network for further extensive research.

## 1. Introduction

Gram-negative bacterium *L. pneumophila* was identified in 1976 at the annual convention of American legion, which caused a serious pneumophila, resulting in a lethality rate of 15.9% [1]. It has been reported that the pathogenic bacteria *L. pneumophila* has a versatile arsenal of effectors, keeping its virulence by expressing over 330 individual effectors through the Dot/Icm secretion system [2,3]. Moreover, further studies of pathogenic strategies revealed that after entering the cytoplasm of the host cell, the bacterium avoids its lysosomal-mediated degradation by escaping the endosomal-trafficking pathway and establishes *Legionella*-containing vacuoles (LCV) [4]. These specialized membrane-bound organelles are rich in nutrients and without lysosome hydrolases, providing *Legionella* with an ideal environment for its intracellular replication [5]. During the formation of LCVs, many post-translational modifications (PTMs) are involved, removing chemical moieties from protein residues, or attaching modifying groups to target protein, which mediates numerous physiological processes by their unique biochemical activities. Up to now, over 400 different types of PTMs have been identified in eukaryotic cells such as phosphorylation, glycosylation, acetylation, ADP-ribosylation and ubiquitination [6,7,8]. Among these, ubiquitination, a ubiquitous post-translational modification, which regulates a variety of physiological processes in eukaryotic cells, such as protein homeostasis, cell cycle, immune response, DNA repair and vesicle transport, has been studied for several decades [9].

The function and mechanism of canonical ubiquitination has been already well established. It occurs through a series of enzymatic reactions. First, the ubiquitin activating E1 consumes ATP and activates the C-terminal carboxyl group of ubiquitin and forms a thioester bond with cysteine at the active site of ubiquitin conjugating enzyme E2. Then, ubiquitin ligase E3 transfers ubiquitin from E2-Ub to a specific substrate. Finally, an isopeptide bond is formed between the carboxyl group of glycine at position 76 of ubiquitin and the ε-amino group of Lys or the α-amino group of Met1 of a substrate protein [10,11]. Intriguingly, owing to the key role of ubiquitination in the life of eukaryotic cells, many pathogens have derived a series of effector proteins targeting the host ubiquitination process during the long-term evolution with host cells, to construct a conducive internal environment for the reproduction of pathogens [12,13].

*L. pneumophila* as the pathogen causing pneumonia, also derived numerous effector proteins to modulate host ubiquitination and the most striking example of these to date is the SidE effector family [14,15,16]. The SidE family contains four highly conserved members SidE, SdeA, SdeB, and SdeC that mediate a noncanonical ubiquitination system to facilitate the optimal *Legionella* virulence. While the importance and the inherent understanding of canonical ubiquitination has been known for a long time, the atypical ubiquitination catalyzed by the SidE family shows an unprecedented aspect in this area, which has attracted a lot of attention. Here we will review the recent progress regarding the mechanism and modulation of SidE family effectors and the pathogenic strategies of *L. pneumophila* related to this ubiquitination process.

## 2. SidE Family Effectors Catalyze a Non-Canonical Ubiquitination Process

**Figure 1:**
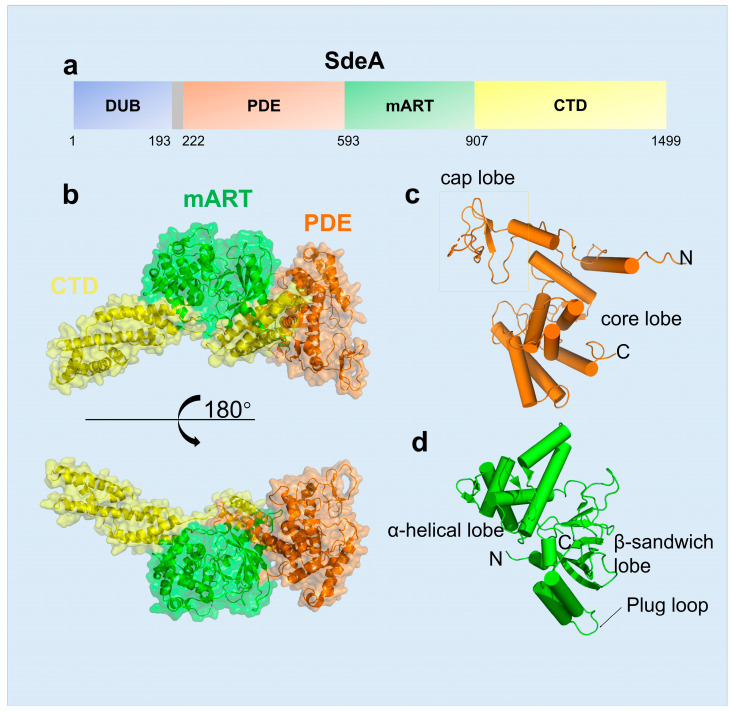
Overall structure of SdeA. (**a**) Domain diagram of SdeA (1–1499), SdeA contains four domains, DUB (blue), PDE (orange-yellow), mART (green) and CTD (golden-yellow), from N-terminus to C-terminus. (**b**) Two views of overall structure of SdeA (231–1190) colored as in a. (**c**) Structure of SdeA PDE domain. (**d**) Structure of SdeA mART domain, α-helical lobe and β-sandwich lobe are marked, and the “plug loop” was also labeled.

The non-canonical ubiquitination by SidE family proteins differs from the canonical ubiquitination system in several aspects, including structure characteristics of enzymes, energy consumption and the number of reaction enzymes or steps. Firstly, for structural features, SidE family members are large proteins (approximately 1500 residues), which contain a DUB (deubiquitinase) domain, a PDE (phosphodiesterase) domain, an mART (mono-ADP ribosyltransferase) domain and a C-terminal domain (CTD) (Figure 1a,b) [17,18]. Secondly, for the energy source, nicotinamide adenine dinucleotide (NAD^+^) is required by the mART domain of SidE, which is a putative catalytic motif typically found in bacterial toxins [19,20]. Thirdly, this non-canonical ubiquitination is catalyzed only by one protein in an all-in-one mode rather than the three steps mode of canonical ubiquitination. Finally, Arg42 of ubiquitin and primarily a serine residue of substrate are linked by a phosphoribosyl moiety, so this type of ubiquitination is also named PR (phosphoribosyl)-ubiquitination. Recent studies also found that SdeC-mediated PR-ubiquitination also modifies tyrosine residues in host proteins [21].

### 2.1. The Structural Features of SidE Family

The SidE family protein contains four domains, including DUB, mART, PDE and CTD domains, and each one has its independent function or regulates another. The DUB domain, comprising ~200 residues in the N-terminus of SidE, was first characterized to have deubiquitinase activity, with a preference for Lys-63 Linked polyubiquitin chains [22]. The PDE domain spans residues approximately 200–600, which is formed by two lobes: a larger helical core lobe containing the catalytic pocket and a smaller cap lobe covering from the top [23] (Figure 1c). Structural comparison revealed that human SAMHD1, the dNTP hydrolase related to innate immune response, is the closest structural homologue in mammals of SdeA [24]. The SdeA mART domain contains a typical Rossmann fold and shows the conserved characteristics among all known mART toxins in bacteria [19]. Two-lobe structures constitute the SdeA mART domain, one with an N-terminal α-helical lobe and the other with a C-terminal β-sandwich lobe (Figure 1d). Even though the similarity between SdeA mART and other mART toxins exist, there are some weak differences in structural details. For example, the PN loop and ARTT loop in the mART domain are different from those of other mART proteins. Moreover, the plug loop, two consecutive helices connected by a loop, inserts into and interacts with the PDE domain, which is related to the activity of mART but not to the PDE domain [15].

### 2.2. The Novel-Ubiquitination Machinery of the SidE Family

**Figure 2:**
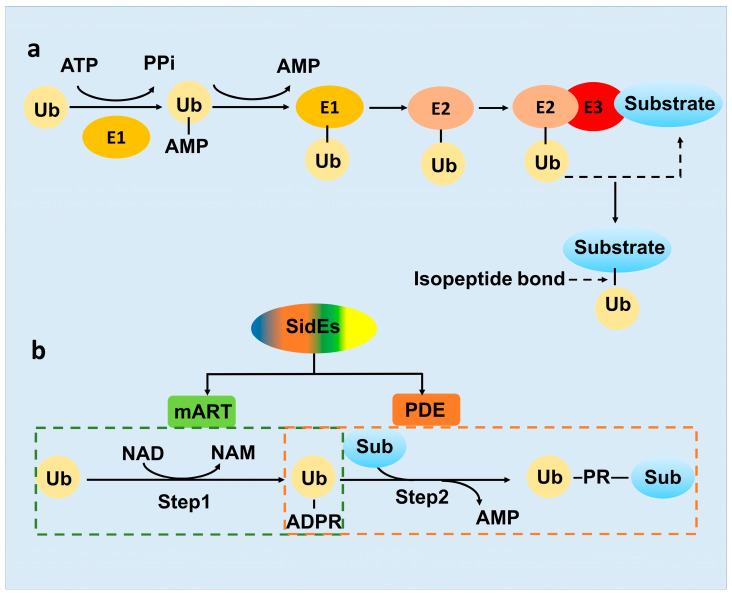
The mechanism diagram of E1-E2-E3 conventional ubiquitination and SidEs PR-ubiquitination. (**a**) Ubiquitin activating enzyme E1, ubiquitin conjugation enzyme E2 and ubiquitin ligase E3 are working together for the conventional type of ubiquitination. (**b**) SidE family proteins could catalyze the whole PR-ubiquitination reaction by itself, mART and PDE domain involved the first and second step respectively. NAM, nicotinamide.

As mentioned above, ubiquitination as an important protein PTM, was well studied for decades [25]. However, in 2016, SdeA protein in *L. pneumophila* was identified to be capable of performing a non-canonical ubiquitination by itself [20]. In contrast to the conventional ATP-driving E1-E2-E3 cascade (Figure 2a), the ubiquitination catalyzed by SdeA effector requires NAD^+^ as energy [26]. Overall, it is strikingly different between the three-enzyme systems and the all-in-one ubiquitination machinery SidE. While SidE family protein comprises four domains, only the enzymatic activities of the PDE and mART domain are involved in the ubiquitination process. The SidE ligase machinery was divided into two distinct parts, Ub activation and Ub-substrate ligation, which was catalyzed by the mART and PDE domain respectively (Figure 2b) [26].

First, the mART domain exhibits ADP-ribosyl transferase activity, using nucleotide cofactor NAD^+^ as energy, leading to ADP-ribose group covalently added to Arg42 of Ub forming ADPR-Ub [26] (Figure 2b). ADP-ribosylation is also one of the most important types of protein PTMs, discovered in bacterial pathogen *Corynebacterium diphtheria* originally and in the eukaryotic cell subsequently, which regulate various cellular processes, including tumorigenesis and DNA repair [27,28]. Despite that ART protein Parp9 interacts with the E3 ligase Dtx3L to add mono-ADP-ribose group to the carboxyl terminus of ubiquitin molecule [29], ADP-ribosylation of ubiquitin catalyzed by SidE mART is also an example of a crosslink between ADP-ribosylation ubiquitination.

Second, the SidE PDE domain recognizes and binds the ADPR-Ub produced by the mART domain, exhibits phosphodiesterase activity to cleave the phosphoanhydride bond in ADPR-Ub and produce phosphoribosylated ubiquitin (PR-Ub) [23]. Meanwhile, in the presence of a substrate protein, the SdeA PDE domain utilizes a substrate binding cleft (constituted by N404, Q405, M408, L411 and S428), juxtaposed with the catalytic site, to position serines of the substrates for ubiquitination. During the reaction, a transient SdeA H277-PR-Ub intermediate was first formed and subsequently nucleophilic attacked by the OH group of the target serine of the substrate. Finally, PR-Ub was transferred to serine residues in target proteins, with the release of AMP [30] (Figure 2b). The PDE domain in the SidE family protein shares ~23% sequence identity with their closest similarity protein PA4781 from *Pseudomonas aeruginosa* and possesses the conserved catalytic residues, H277-H407-E340 catalytic triad. The reaction catalyzed by the PDE domain is similar to a phosphotransferase activity and akin in part to the activity of His kinases [31,32]. Notably, ADPR-Ub can be produced by the SdeA PDE mutant (H277A) and PR-Ub can still be transferred to a target protein, if the SdeA mART domain truncation was supplied with ADPR-Ub as a substrate, suggesting that these two reactions were separable [15,26,33].

## 3. Activity of the SidE Family Was Strictly Modulated by Many Effectors

Physiological processes in eukaryotic or prokaryotic cells are influenced and modulated extensively by other molecules, including chemical substances and proteins. Similarly, the activity of SidE family proteins is also strictly controlled by other proteins. Recently, *L. pneumophila* effectors, SidJ, SdjA, DupA and DupB have been proved to regulate the activity of SidE family proteins by some novel modes.

### 3.1. SidJ Interacts with Calmodulin to Modify SdeA

**Figure 3:**
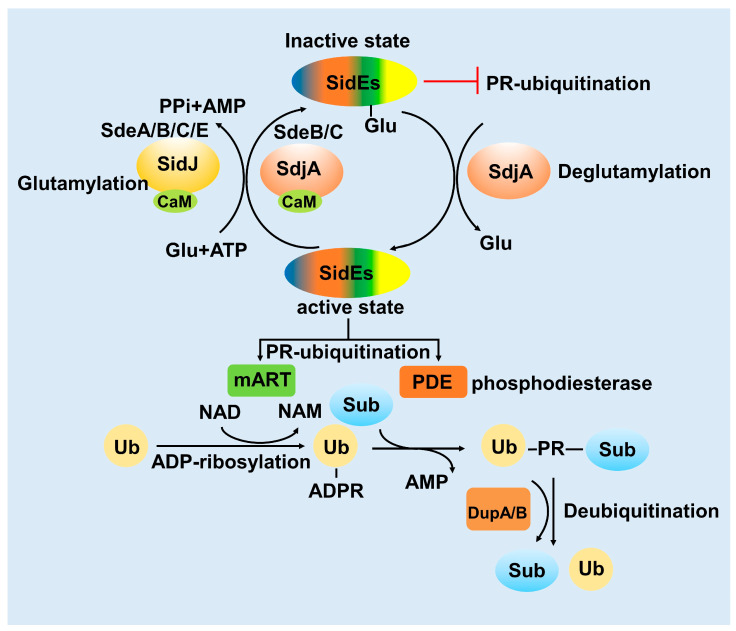
The modulation model of SidE family proteins. SidEs catalyze PR-ubiquitination by their mART and PDE domains. SidJ-CaM and SdjA-CaM mediate glutamylation to inactivate SidEs (SidJ-CaM inactivate SdeA/B/C/E, SdjA-CaM inactivate SdeB/C). The inactivated SidEs-Glu could be reactivated again by SdjA, named deglutamylation. The PR-ubiquitination substrates could be cleaved by DupA/B, releasing substrates and Ub again.

The ubiquitination activity of SdeA has a relatively strong toxic effect on host cells. However, this excessive toxic effect is not conducive to the proliferation of *L. pneumophila*. In 2015, the *L. pneumophila* effector protein SidJ was found to inhibit the toxicity of SdeA in the host [34]. In the subsequent study, it was proved that SidJ suppresses the ubiquitination activity of SdeA in vivo [35]. However, it was still unknown why SidJ can inhibit the activity of SidE family proteins only in the host cell at that time. In the process of exploring this question, calmodulin (CaM), the Ca^2+^ binding protein in eukaryotic cells, appears to participate in the regulation of SdeA by interacting with the *L. pneumophila* effector protein SidJ. Then, four independent studies revealed that SidJ and calmodulin form a stable complex, catalyzing a distinct PTM to the key catalytic residues of the SidE family protein, turning SidE into the “inactive state” (Figure 3). This unusual PTM was polyglutamylation and the exactly modified residue of SdeA was E860, a key catalytic residue in the mART domain [36,37,38,39]. The discovery of SidJ as a CaM-activated glutamylase explained that how SidJ-CaM complex inactivates the SidE family protein. However, there are still several intriguing questions to be further explored. First, for the mechanism details about CaM dependent activating mode, Sulpizio et.al., proposed that CaM-binding may stabilize the activation loop, which is vital for protein kinases, in an activated state via the CaM N-loop [39]. Second, for the substrate specificity of SidJ, it remains not fully understood whether SidJ only targets the SidE family protein. Bhogaraju et al., found that glutamylation signals still remained when the host cell was infected by *Legionella* strains lacking SidE family genes, indicating that the SidE family protein might not be the only substrates of SidJ-CaM. This finding was striking and interesting in that the pathogenic bacteria effectors along with the eukaryotic host protein might modify another effector together [37].

### 3.2. SdjA Reverses the Glutamylation Modification of SdeA

Remarkably, the modification mode of SidJ-CaM towards SdeA unveils an archetypal example that the pathogenic bacterial effector protein catalyzes glutamylation, modulating the PR-ubiquitination mediated by the SidE family protein in the host cell [40]. E860 is the key catalytic residue of the SdeA mART domain, which is polyglutamated by the SidJ-CaM complex, indicating that SidJ-CaM displays specificity towards this residue. Furthermore, Vincent et al., solved the cryo-EM structure of SdeA-SidJ-CaM intermediate complexes, proving that the kinase-like site of SidJ adenylates the active-site Glu in SidE in the presence of ATP and Mg^2+^, forming a stable intermediate complex. At the same time, the insertion loop in the active site of the SidJ kinase domain accommodates the donor Glu near the acyl-adenylates site, facilitating the reaction of glutamylation [41].

Furthermore, based on the fact that several *Legionella* or other bacteria effectors are working together to regulate one physiological process, we wondered whether other effectors also participate in the regulation of SidEs. Interestingly, SdjA, a paralog protein of SidJ which shows 57% sequence identity [42], shows glutamylation activity to SdeB and SdeC but not SdeA. Moreover, SdjA cannot complement the virulence defects displayed by a mutant lacking SidJ [43]. Due to the unusual characteristic of SdjA compared with SidJ, the function of effector SdjA still remains unknown. Coincidentally, two *Legionella* homologous effector proteins MavC and MvcA, have been proved to work together to stimulate and antagonize another unconventional ubiquitination, respectively [14,44,45]. In this distinct type of ubiquitination, MavC catalyzes the attachment of Ub to UBE2N by its transglutaminase activity (termed ubiquitination), while MvcA catalyzes the opposite process releasing ubiquitin from Ub-UBE2N by its deamidase activity (Deubiquitination) [14,16,45]. Interestingly, we found that SdjA contains deglutamylase activity, changing SdeA-Glu into SdeA in the absence of CaM, thereby antagonizing the activity of SidJ. Actually, SdjA was a bifunctional enzyme that exhibits distinct activities towards SidE family proteins and the specificity was dependent on its N-terminal region (Figure 3) [43].

### 3.3. DupA and DupB Function as Deubiquitinases for PR-Ubiquitination

Conventional ubiquitination is a reversible process, the substrate of ubiquitination can be re-cleaved into ubiquitin and substrate by deubiquitinating enzymes [46]. The novel ubiquitination mediated by the SidE-effector proteins involves the formation of thioester bonds between substrates serine hydroxyl and ubiquitin [26]. In this process, the PDE domain of SidE can cleave ADPR-Ub to generate AMP and PR-Ub in the absence of substrates [26]. Interestingly, the *L. pneumophila* effectors DupA and DupB, two homologous proteins of the PDE domain with 70% sequence similarity to SdeA PDE, have been proved to exhibit activity to process ADPR-Ub to PR-Ub [47]. So that, the balance of PR-ubiquitination of multiple substrates in the host cell was controlled by these two specific deubiquitinases upon bacterial infection stringently. While SidEs catalyze PR-ubiquitination with its PDE domain in the second step, DupA and DupB catalyze deubiquitination also via their PDE domains [48]. This is reminiscent of the characteristics of SidJ/SdjA, or MavC/MvcA, which were mentioned above (Figure 3).

## 4. Multiple Host Proteins Targeted by SidE Family Effectors

Previous studies indicated that the host substrates of the SidE family are related with the endoplasmic reticulum (ER) and Golgi in the host cell, resulting in disturbances of their transport pathways, which modulates the internal host environment and promotes the formation of LCV. However, along with the deepening of studies into the biological significance of SidE-mediated ubiquitination, especially the use of DupA/B deletion bacterial strain, other physiological systems, such as endo-lysosomal system, mitochondria, proteasomal subunits, cytoskeleton and nuclear membrane related proteins, have also been reported to be regulated by this ubiquitination [48]. It is necessary to determine the exact relationship between the ubiquitination catalyzed by SidE and these cellular processes to cast light on how *L. pneumophila* exploits these effectors for survival and proliferation.

### 4.1. The Effects of SidE Family Proteins on Endoplasmic Reticulum

*L. pneumophila* is an intravacuolar pathogen, utilizing a type IVB secretion system (T4SS) to translocate effector proteins into the host cytosol to establish LCV, an endoplasmic reticulum (ER)-associated organelle [49,50]. However, these bacterial effector proteins are unable to form an LCV themselves which means that they need to make use of the substances or protein substrates in the host cell for this process. Endoplasmic reticulum is a continuous omental system, a cystic, vesicular, and tubular structure organelle formed by a single membrane, which is in charge of the production and movement of proteins and other molecules [51]. Endoplasmic reticulum could be classified into the perinuclear, ribosome-associated ER sheet and tubular ER and the tubular ER is a vast network of cylinders that are enriched with some structural ER membrane proteins, such as reticulon family proteins [52]. Previous studies identified that several ER-associated GTPases and reticulon 4 (Rtn4) are PR-ubiquitinated by SidE family proteins. During its infection, *L. pneumophila* exploits effectors to regulate the dynamics of membranes to create LCV. PR-ubiquitination was utilized by *Legionella* to modify ER-related proteins, such as RTN3, RTN4, TEX264, FAM134A, FAM134B and FAM134C, giving rise to ER membrane fragmentation and dynamic defect [48,53,54]. Among these ER-related proteins, RTN4 is required to induce the formation and stabilization of endoplasmic reticulum tubules, regulating membrane morphogenesis in the ER [55], and previously regarded as a critical substrate for the formation of LCV. FAM134 family proteins (FAM134A, FAM134B and FAM134C) are ER-anchored autophagy receptors, which mediate ER transports into lysosome, promoting membrane remodeling and ER dissociation. Furthermore, FAM134B targets the ER fragments into autophagosomes via interaction with ATG8 family proteins [54]. Taken together, these suggest that SidE family proteins mediated PR-ubiquitination of host substrates to affect the normal function of endoplasmic reticulum (Table 1).

### 4.2. The Effects of SidE Family Proteins on the Golgi Complex

In the early stage of infection, *L. pneumophila* exploits effectors, such as SidE family proteins, to manipulate Rab1 and other ER-related proteins to intercept the versicles to the LCV [68]. Actually, the downstream process after ER vesicles fusing to the Golgi complex is also disturbed. Recently, the relationship between PR-ubiquitination and the Golgi complex has received increasing attention. The Golgi complex, the cystic structure apparatus formed by the elementary membrane, is the component of the eukaryotic endomembrane system, which functions as the PTM factory for protein modification, classification and translocation [69]. The vesicles from the endoplasmic reticulum could fuse with the Golgi membrane, delivering the inclusions into the Golgi lumen, where the newly synthesized peptide chains continue to complete their modification and packing [70].

**Figure 4:**
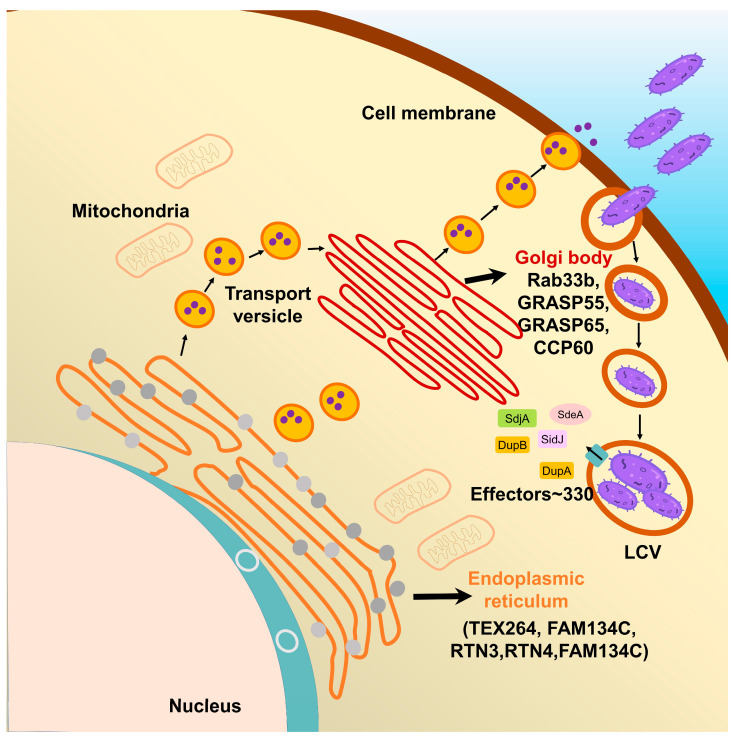
The spatial relationship between LCV, ER and Golgi and the localization of the various SidE target proteins.

Most obviously, compared with the relative comprehensive understanding that *L. pneumophila* markedly disrupts the ER trafficking pathway, it is elusive how the SidE family proteins affect the function of the Golgi apparatus and which Golgi-related proteins in the host cell are taking part in the PR-ubiquitination. Notably, Shin et al., showed that two deubiquitinases (DupA and DupB) specifically cleave PR-ubiquitin from serine on substrates and take advantage of the catalytically inactive DupA and its affinity for PR-ubiquitinated protein to capture and identify nearly 180 host proteins targeted by SidE family proteins [48]. Among these substrates, some Golgi-related proteins were also identified, such as GRASP55, TMED8, GCP60, YIF1A, RAB33B and SNX5. Notably, GRASP55 and GCP60 had the highest ratio among these Golgi protein substrates (Figure 4, Table 1). 

GRASP55 plays a vital role in the maintenance of the Golgi integrality [64,65]. GRASP65 and GRASP55 are homologous proteins, both belong to the same protein family named GRASP, which function in the connection of the Golgi stack and the maintenance of the Golgi structure through self-interaction and interactions with other Golgi proteins [71]. They are localized to Golgi cisternae and required for the ER-to-Golgi transport of specific cargo, which contains C-terminal valine motif [72]. It has been known that the activities of mammalian GRASPs are regulated by serine phosphorylation, one of the most canonical PTMs, resulting in Golgi fragmentation [73]. Recently, Liu et al., confirmed that the C-terminus of SdeA is not only critical for its Golgi localization, but also for its ability to PR-ubiquitinate Golgi protein in the host cell. Taken together, the PR-ubiquitination of GRASP65 and GRASP55 by SidE family proteins, causes disruption of Golgi integrity, thus preventing their ability to aggregate and form oligomeric states. In fact, the presence or absence of PR-ubiquitination of GRASPs can have an important impact on the host secretory pathway, while is not linked directly to the recruitment of Golgi membranes to the growing LCV [74].

## 5. Conclusions

In this review, we summarize the mechanisms, modulation and protein substrates related to endoplasmic reticulum and Golgi apparatus of non-canonical ubiquitination by SidE family proteins during the pathogenesis of *L. pneumophila*. Harboring three enzymatic activities, SidE family proteins also undergo extensive modulations. In terms of activity modulation, the important role of calmodulin and the need to study the structure and function of SdjA are emphasized. Regarding substrates, we summarized mainly the substrates related to endoplasmic reticulum and Golgi apparatus, which have been studied more extensively at present, and pointed out the perspectives for subsequent research on substrates of other physiological processes.

Specifically, SidJ inactivates SdeA and SdjA renders SdeA to regain its activity of PR-ubiquitination [43]. This SdeA regulation mode is associated with calmodulin, the calcium binding protein in eukaryotic cells, which participates in many physiology processes and especially plays a vital role in the calcium signal transduction pathway [75]. However, it still remains unknown whether calmodulin is only used by *L. pneumophila* to control its virulence of SidE family effectors or is simultaneously influencing other physiology processes involved in signal transduction. This will be interesting to be investigated by future studies. Moreover, pathogens need to strictly control their virulence to proliferate normally. From the aspect of host-pathogen interaction, when the host cell was infected by pathogens, they also need to evolve approaches to counteract the influence of pathogens. Therefore, it is an interesting question whether the need of calmodulin binding for the activity of SidJ in *L. pneumophila* is a beneficial approach for the pathogen to modulate the activity of its effectors or a strategy exploited by the host to inactivate the toxic effectors of pathogens. Based on the recent study, SdjA seems to eb a critical member in the regulation network of the SidE family and its deglutamylation activity was not dependent on calmodulin binding. However, the key domains and residues for deglutamylation activity in SdjA still need further investigation [43].

With a growing number of PR-ubiquitination substrates identified, more and more related physiological processes have been found. This means that the functions of SidE family effectors are more complicated and significant for the pathogen than what we have ever known. Whereas some advances of PR-ubiquitination in endoplasmic reticulum and the Golgi complex have been achieved, the effects on other related processes, such as the Endo-lysosomal system, mitochondria, proteasomal subunits, cytoskeleton and nuclear membrane related proteins, still need to be further explored. Moreover, temporal and spatial regulation of the activity of SidEs by the modulation effectors in these physiological processes will also be interesting subjects in future studies.

## Figures and Tables

**Table 1:** List of SidEs and their major related proteins in this review.

Gene ID	Species	Aliases	Function	Reference
lpg2157	*L. pneumophila*	SdeA	PR-ubiquitination	[20]
lpg2156	*L. pneumophila*	SdeB	PR-ubiquitination	
lpg2153	*L. pneumophila*	SdeC	PR-ubiquitination	
lpg0234	*L. pneumophila*	SidE	PR-ubiquitination	
lpg2155	*L. pneumophila*	SidJ	Inhibits SdeA, SdeB, SdeC and SidE ubiquitinating activity by Glutamylation.	[35,36,38]
lpg2508	*L. pneumophila*	SdjA	Reverses the SidJ-CaM modification of SdeA.	[43]
lpg2154	*L. pneumophila*	DupA	Regulates Phosphoribosyl-Linked Serine Ubiquitination by Deubiquitination.	[47,48]
lpg2509	*L. pneumophila*	DupB		
10313	Homo sapiens	RTN3	Induces the formation of ER	[56]
57142	Homo sapiens	RTN4	Stabilization of endoplasmic reticulum (ER)	[55,56,57]
162427	Homo sapiens	FAM134C	Endoplasmic reticulum remodeling, ER-phagy, and Collagen quality control.	[58]
51368	Homo sapiens	TEX264	ATG8-Interacting Protein Critical for ER Remodeling.ER-phage.	[59,60]
83452	Homo sapiens	Rab33b	ER-associated Rab small GTPases.Regulators of Golgi homeostasis and trafficking.	[61,62]
26003	Homo sapiens	GRASP55	Function in the connection of Golgi stack and the maintenance of Golgi structure	[63,64]
64689	Homo sapiens	GRASP65		
64746	Homo sapiens	GCP60	Affecting protein transport between the endoplasmic reticulum and Golgi.	[65]
10897	Homo sapiens	YIF1A	Role in transport between endoplasmic reticulum and Golgi.	[66]
27131	Homo sapiens	SNX5	Mediates retrograde transport of cargo proteins from endosomes to the trans-Golgi network.	[67]

Notes: this table only contains a small number of ER and Golgi related substrates.

## Data Availability

No new data were created or analyzed in this study. Data sharing is not applicable to this article.

## References

[1] Fraser D.W., Tsai T.R., Orenstein W., Parkin W.E., Beecham H.J., Sharrar R.G., Harris J., Mallison G.F., Martin S.M., Mcdade J.E. (1977). Legionnaires’ Disease. N. Engl. J. Med..

[2] Luo Z.Q., Isberg R.R. (2004). Multiple substrates of the Legionella pneumophila Dot/Icm system identified by interbacterial protein transfer. Proc. Natl. Acad. Sci. USA.

[3] Ensminger A.W. (2016). Legionella pneumophila, armed to the hilt: Justifying the largest arsenal of effectors in the bacterial world. Curr. Opin. Microbiol..

[4] Isberg R.R., O’connor T.J., Heidtman M. (2009). The Legionella pneumophila replication vacuole: Making a cosy niche inside host cells. Nat. Rev. Microbiol..

[5] Robinson C.G., Roy C.R. (2006). Attachment and fusion of endoplasmic reticulum with vacuoles containing Legionella pneumophila. Cell. Microbiol..

[6] Ramazi S., Zahiri J. (2021). Posttranslational modifications in proteins: Resources, tools and prediction methods. Database.

[7] Mann M., Jensen O.N.J.N.B. (2003). Proteomic analysis of post-translational modifications. Nat. Biotechnol..

[8] Olsen J.V., Mann M.J.M., Mcp C.P. (2013). Status of Large-scale Analysis of Post-translational Modifications by Mass Spectrometry. Mol. Cell. Proteom..

[9] Sun S.C. (2008). Deubiquitylation and regulation of the immune response. Nat. Rev. Immunol..

[10] Hershko A., Ciechanover A. (1998). The ubiquitin system. Annu. Rev. Biochem..

[11] Zheng N., Shabek N. (2017). Ubiquitin Ligases: Structure, Function, and Regulation. Annu. Rev. Biochem..

[12] Yan F., Huang C., Wang X., Tan J., Cheng S., Wan M., Wang Z., Wang S., Luo S., Li A. (2020). Threonine ADP-Ribosylation of Ubiquitin by a Bacterial Effector Family Blocks Host Ubiquitination. Mol. Cell.

[13] Lin Y., Hu Q., Zhou J., Yin W., Yao D., Shao Y., Zhao Y., Guo B., Xia Y., Chen Q. (2021). Phytophthora sojae effector Avr1d functions as an E2 competitor and inhibits ubiquitination activity of GmPUB13 to facilitate infection. Proc. Natl. Acad. Sci. USA.

[14] Gan N., Nakayasu E.S., Hollenbeck P.J., Luo Z.Q. (2019). Legionella pneumophila inhibits immune signalling via MavC-mediated transglutaminase-induced ubiquitination of UBE2N. Nat. Microbiol..

[15] Dong Y., Mu Y., Xie Y., Zhang Y., Han Y., Zhou Y., Wang W., Liu Z., Wu M., Wang H. (2018). Structural basis of ubiquitin modification by the Legionella effector SdeA. Nature.

[16] Mu Y., Wang Y., Huang Y., Li D., Han Y., Chang M., Fu J., Xie Y., Ren J., Wang H. (2020). Structural insights into the mechanism and inhibition of transglutaminase-induced ubiquitination by the Legionella effector MavC. Nat. Commun..

[17] Bardill J.P., Miller J.L., Vogel J.P. (2005). IcmS-dependent translocation of SdeA into macrophages by the Legionella pneumophila type IV secretion system. Mol. Microbiol..

[18] Krissinel E., Henrick K. (2007). Inference of macromolecular assemblies from crystalline state. J. Mol. Biol..

[19] Han S., Arvai A.S., Clancy S.B., Tainer J.A. (2001). Crystal structure and novel recognition motif of Rho ADP-ribosylating C3 exoenzyme from Clostridium botulinum: Structural insights for recognition specificity and catalysis1 1Edited by D. Rees. J. Mol. Biol..

[20] Qiu J., Sheedlo M.J., Yu K., Tan Y., Nakayasu E.S., Das C., Liu X., Luo Z.Q. (2016). Ubiquitination independent of E1 and E2 enzymes by bacterial effectors. Nature.

[21] Zhang M., Mcewen J.M., Sjoblom N.M., Kotewicz K.M., Isberg R.R., Scheck R.A. (2021). Members of the Legionella pneumophila Sde family target tyrosine residues for phosphoribosyl-linked ubiquitination. RSC Chem. Biol..

[22] Sheedlo M.J., Qiu J., Tan Y., Paul L.N., Luo Z.Q., Das C. (2015). Structural basis of substrate recognition by a bacterial deubiquitinase important for dynamics of phagosome ubiquitination. Proc. Natl. Acad. Sci. USA.

[23] Wang Y., Shi M., Feng H., Zhu Y., Liu S., Gao A., Gao P. (2018). Structural Insights into Non-canonical Ubiquitination Catalyzed by SidE. Cell.

[24] Ji X., Wu Y., Yan J., Mehrens J., Yang H., Delucia M., Hao C., Gronenborn A.M., Skowronski J., Ahn J. (2013). Mechanism of allosteric activation of SAMHD1 by dGTP. Nat. Struct. Mol. Biol..

[25] Varshavsky A. (1997). The ubiquitin system. Trends Biochem. Sci..

[26] Bhogaraju S., Kalayil S., Liu Y., Bonn F., Colby T., Matic I., Dikic I. (2016). Phosphoribosylation of Ubiquitin Promotes Serine Ubiquitination and Impairs Conventional Ubiquitination. Cell.

[27] Honjo T., Nishizuka Y., Hayaishi O., Kato I. (1968). Diphtheria Toxin-dependent Adenosine Diphosphate Ribosylation of Aminoacyl Transferase II and Inhibition of Protein Synthesis. J. Biol. Chem..

[28] Corda D., Di Girolamo M. (2003). Functional aspects of protein mono-ADP-ribosylation. EMBO J..

[29] Yang C.S., Jividen K., Spencer A., Dworak N., Ni L., Oostdyk L.T., Chatterjee M., Kuśmider B., Reon B., Parlak M. (2017). Ubiquitin Modification by the E3 Ligase/ADP-Ribosyltransferase Dtx3L/Parp9. Mol. Cell.

[30] Kotewicz K.M., Ramabhadran V., Sjoblom N., Vogel J.P., Haenssler E., Zhang M., Behringer J., Scheck R.A., Isberg R.R. (2017). A Single Legionella Effector Catalyzes a Multistep Ubiquitination Pathway to Rearrange Tubular Endoplasmic Reticulum for Replication. Cell Host Microbe.

[31] Kalayil S., Bhogaraju S., Bonn F., Shin D., Liu Y., Gan N., Basquin J., Grumati P., Luo Z.-Q., Dikic I. (2018). Insights into catalysis and function of phosphoribosyl-linked serine ubiquitination. Nature.

[32] Klumpp S., Krieglstein J. (2002). Phosphorylation and dephosphorylation of histidine residues in proteins. Eur. J. Biochem..

[33] Akturk A., Wasilko D.J., Wu X., Liu Y., Zhang Y., Qiu J., Luo Z.-Q., Reiter K.H., Brzovic P.S., Klevit R.E. (2018). Mechanism of phosphoribosyl-ubiquitination mediated by a single Legionella effector. Nature.

[34] Havey J.C., Roy C.R. (2015). Toxicity and SidJ-Mediated Suppression of Toxicity Require Distinct Regions in the SidE Family of Legionella pneumophila Effectors. Infect. Immun..

[35] Qiu J., Yu K., Fei X., Liu Y., Nakayasu E.S., Piehowski P.D., Shaw J.B., Puvar K., Das C., Liu X. (2017). A unique deubiquitinase that deconjugates phosphoribosyl-linked protein ubiquitination. Cell Res..

[36] Black M.H., Osinski A., Gradowski M., Servage K.A., Pawłowski K., Tomchick D.R., Tagliabracci V.S. (2019). Bacterial pseudokinase catalyzes protein polyglutamylation to inhibit the SidE-family ubiquitin ligases. Science.

[37] Bhogaraju S., Bonn F., Mukherjee R., Adams M., Pfleiderer M.M., Galej W.P., Matkovic V., Lopez-Mosqueda J., Kalayil S., Shin D. (2019). Inhibition of bacterial ubiquitin ligases by SidJ-calmodulin catalysed glutamylation. Nature.

[38] Gan N., Zhen X., Liu Y., Xu X., He C., Qiu J., Liu Y., Fujimoto G.M., Nakayasu E.S., Zhou B. (2019). Regulation of phosphoribosyl ubiquitination by a calmodulin-dependent glutamylase. Nature.

[39] Sulpizio A., Minelli M.E., Wan M., Burrowes P.D., Wu X., Sanford E.J., Shin J.H., Williams B.C., Goldberg M.L., Smolka M.B. (2019). Protein polyglutamylation catalyzed by the bacterial calmodulin-dependent pseudokinase SidJ. eLife.

[40] Sulpizio A.G., Minelli M.E., Mao Y. (2019). Glutamylation of Bacterial Ubiquitin Ligases by a Legionella Pseudokinase. Trends Microbiol..

[41] Osinski A., Black M.H., Pawłowski K., Chen Z., Li Y., Tagliabracci V.S. (2021). Structural and mechanistic basis for protein glutamylation by the kinase fold. Mol. Cell.

[42] Liu Y., Luo Z.Q. (2007). The Legionella pneumophila effector SidJ is required for efficient recruitment of endoplasmic reticulum proteins to the bacterial phagosome. Infect. Immun..

[43] Song L., Xie Y., Li C., Wang L., He C., Zhang Y., Yuan J., Luo J., Liu X., Xiu Y. (2021). The Legionella Effector SdjA Is a Bifunctional Enzyme That Distinctly Regulates Phosphoribosyl Ubiquitination. mBio.

[44] Valleau D., Quaile A.T., Cui H., Xu X., Evdokimova E., Chang C., Cuff M.E., Urbanus M.L., Houliston S., Arrowsmith C.H. (2018). Discovery of Ubiquitin Deamidases in the Pathogenic Arsenal of Legionella pneumophila. Cell Rep..

[45] Gan N., Guan H., Huang Y., Yu T., Fu J., Nakayasu E.S., Puvar K., Das C., Wang D., Ouyang S. (2020). Legionella pneumophila regulates the activity of UBE2N by deamidase-mediated deubiquitination. EMBO J..

[46] Komander D., Clague M.J., Urbé S. (2009). Breaking the chains: Structure and function of the deubiquitinases. Nat. Rev. Mol. Cell Biol..

[47] Wan M., Sulpizio A.G., Akturk A., Beck WH J., Lanz M., Faça V.M., Smolka M.B., Vogel J.P., Mao Y. (2019). Deubiquitination of phosphoribosyl-ubiquitin conjugates by phosphodiesterase-domain-containing Legionella effectors. Proc. Natl. Acad. Sci. USA.

[48] Shin D., Mukherjee R., Liu Y., Gonzalez A., Bonn F., Liu Y., Rogov V.V., Heinz M., Stolz A., Hummer G. (2020). Regulation of Phosphoribosyl-Linked Serine Ubiquitination by Deubiquitinases DupA and DupB. Mol. Cell.

[49] Rowbotham T.J. (1980). Preliminary report on the pathogenicity of Legionella pneumophila for freshwater and soil amoebae. J. Clin. Pathol..

[50] Swanson M.S., Isberg R.R. (1995). Association of Legionella pneumophila with the macrophage endoplasmic reticulum. Infect. Immun..

[51] Schwarz D.S., Blower M.D. (2016). The endoplasmic reticulum: Structure, function and response to cellular signaling. Cell. Mol. Life Sci. CMLS.

[52] Nixon-Abell J., Obara C.J., Weigel A.V., Li D., Legant W.R., Xu C.S., Pasolli H.A., Harvey K., Hess H.F., Betzig E. (2016). Increased spatiotemporal resolution reveals highly dynamic dense tubular matrices in the peripheral ER. Science.

[53] Grumati P., Morozzi G., Hölper S., Mari M., Harwardt M.I., Yan R., Müller S., Reggiori F., Heilemann M., Dikic I. (2017). Full length RTN3 regulates turnover of tubular endoplasmic reticulum via selective autophagy. eLife.

[54] Khaminets A., Heinrich T., Mari M., Grumati P., Huebner A.K., Akutsu M., Liebmann L., Stolz A., Nietzsche S., Koch N. (2015). Regulation of endoplasmic reticulum turnover by selective autophagy. Nature.

[55] Wang S., Tukachinsky H., Romano F.B., Rapoport T.A. (2016). Cooperation of the ER-shaping proteins atlastin, lunapark, and reticulons to generate a tubular membrane network. eLife.

[56] Urade T., Yamamoto Y., Zhang X., Ku Y., Sakisaka T. (2014). Identification and characterization of TMEM33 as a reticulon-binding protein. Kobe J. Med. Sci..

[57] Yamamoto Y., Yoshida A., Miyazaki N., Iwasaki K., Sakisaka T. (2014). Arl6IP1 has the ability to shape the mammalian ER membrane in a reticulon-like fashion. Biochem. J..

[58] Reggio A., Buonomo V., Berkane R., Bhaskara R.M., Tellechea M., Peluso I., Polishchuk E., Di Lorenzo G., Cirillo C., Esposito M. (2021). Role of FAM134 paralogues in endoplasmic reticulum remodeling, ER-phagy, and Collagen quality control. EMBO Rep..

[59] An H., Ordureau A., Paulo J.A., Shoemaker C.J., Denic V., Harper J.W. (2019). TEX264 Is an Endoplasmic Reticulum-Resident ATG8-Interacting Protein Critical for ER Remodeling during Nutrient Stress. Mol. Cell.

[60] Chino H., Hatta T., Natsume T., Mizushima N. (2019). Intrinsically Disordered Protein TEX264 Mediates ER-phagy. Mol. Cell.

[61] Starr T., Sun Y., Wilkins N., Storrie B. (2010). Rab33b and Rab6 are functionally overlapping regulators of Golgi homeostasis and trafficking. Traffic.

[62] Nottingham R.M., Ganley I.G., Barr F.A., Lambright D.G., Pfeffer S.R. (2011). RUTBC1 protein, a Rab9A effector that activates GTP hydrolysis by Rab32 and Rab33B proteins. J. Biol. Chem..

[63] Zhang Y., Seemann J. (2021). Rapid degradation of GRASP55 and GRASP65 reveals their immediate impact on the Golgi structure. J. Cell Biol..

[64] Grond R., Veenendaal T., Duran J.M., Raote I., Van Es J.H., Corstjens S., Delfgou L., El Haddouti B., Malhotra V., Rabouille C. (2020). The function of GORASPs in Golgi apparatus organization in vivo. J. Cell Biol..

[65] Sohda M., Misumi Y., Yamamoto A., Yano A., Nakamura N., Ikehara Y. (2001). Identification and Characterization of a Novel Golgi Protein, GCP60, That Interacts with the Integral Membrane Protein Giantin*. J. Biol. Chem..

[66] Jin C., Zhang Y., Zhu H., Ahmed K., Fu C., Yao X. (2005). Human Yip1A specifies the localization of Yif1 to the Golgi apparatus. Biochem. Biophys. Res. Commun..

[67] Van Weering J.R., Sessions R.B., Traer C.J., Kloer D.P., Bhatia V.K., Stamou D., Carlsson S.R., Hurley J.H., Cullen P.J. (2012). Molecular basis for SNX-BAR-mediated assembly of distinct endosomal sorting tubules. EMBO J..

[68] Kawabata M., Matsuo H., Koito T., Murata M., Kubori T., Nagai H., Tagaya M., Arasaki K. (2021). Legionella hijacks the host Golgi-to-ER retrograde pathway for the association of Legionella-containing vacuole with the ER. PLoS Pathog..

[69] Kulkarni-Gosavi P., Makhoul C., Gleeson P.A. (2019). Form and function of the Golgi apparatus: Scaffolds, cytoskeleton and signalling. FEBS Lett..

[70] Li J., Ahat E., Wang Y. (2019). Golgi Structure and Function in Health, Stress, and Diseases. Results Probl. Cell Differ..

[71] Rabouille C., Linstedt A.D. (2016). GRASP: A Multitasking Tether. Front. Cell Dev..

[72] Shorter J., Watson R., Giannakou M.E., Clarke M., Warren G., Barr F.A. (1999). GRASP55, a second mammalian GRASP protein involved in the stacking of Golgi cisternae in a cell-free system. EMBO J..

[73] Feinstein T.N., Linstedt A.D. (2008). GRASP55 regulates Golgi ribbon formation. Mol. Biol. Cell.

[74] Liu Y., Mukherjee R., Bonn F., Colby T., Matic I., Glogger M., Heilemann M., Dikic I. (2021). Serine-ubiquitination regulates Golgi morphology and the secretory pathway upon Legionella infection. Cell Death Differ..

[75] Zhang M., Abrams C., Wang L., Gizzi A., He L., Lin R., Chen Y., Loll Patrick J., Pascal John M., Zhang J.-F. (2012). Structural Basis for Calmodulin as a Dynamic Calcium Sensor. Structure.

